# Ulinastatin alleviates early brain injury after intracerebral hemorrhage by inhibiting necroptosis and neuroinflammation via MAPK/NF-κB signaling pathway

**DOI:** 10.1590/acb370301

**Published:** 2022-05-13

**Authors:** Li Wang, Wei Jiao, Jiayu Wu, Jing Zhang, Min Tang, Yang Chen

**Affiliations:** 1 BS. Anhui Medical University – Wuxi Clinical College – 904th Hospital of Joint Logistic Support Force of PLA – Department of Neurology – Wuxi, China.; 2 BS. Anhui Medical University – Wuxi Clinical College – 904th Hospital of Joint Logistic Support Force of PLA – Department of Nursing – Wuxi, China.; 3 MM. Anhui Medical University – Wuxi Clinical College – 904th Hospital of Joint Logistic Support Force of PLA – Department of Neurology – Wuxi, China.; 4 MM. Anhui Medical University – Wuxi Clinical College – 904th Hospital of Joint Logistic Support Force of PLA – Department of Nursing – Wuxi, China.; 5 MD. Anhui Medical University – Wuxi Clinical College – 904th Hospital of Joint Logistic Support Force of PLA – Department of Neurology – Wuxi, China.

**Keywords:** Cerebral Hemorrhage, Stroke, Brain Injuries, Necrosis, RIP1/RIP3

## Abstract

**Purpose:**

Spontaneous intracerebral hemorrhage (ICH) is a major public health problem with a huge economic burden worldwide. Ulinastatin (UTI), a serine protease inhibitor, has been reported to be anti-inflammatory, immune regulation, and organ protection by reducing reactive oxygen species production, and inflammation. Necroptosis is a programmed cell death mechanism that plays a vital role in neuronal cell death after ICH. However, the neuroprotection of UTI in ICH has not been confirmed, and the potential mechanism is unclear. The present study aimed to investigate the neuroprotection and potential molecular mechanisms of UTI in ICH-induced EBI in a C57BL/6 mouse model.

**Methods:**

The neurological score, brain water content, neuroinflammatory cytokine levels, and neuronal damage were evaluated. The anti-inflammation effectiveness of UTI in ICH patients also was evaluated.

**Results:**

UTI treatment markedly increased the neurological score, alleviate the brain edema, decreased the inflammatory cytokine TNF-α, interleukin‑1β (IL‑1β), IL‑6, NF‑κB levels, and RIP1/RIP3, which indicated that UTI-mediated inhibition of neuroinflammation, and necroptosis alleviated neuronal damage after ICH. UTI also can decrease the inflammatory cytokine of ICH patients. The neuroprotective capacity of UTI is partly dependent on the MAPK/NF-κB signaling pathway.

**Conclusions:**

UTI improves neurological outcomes in mice and reduces neuronal death by protecting against neural neuroinflammation, and necroptosis.

## Introduction

Spontaneous intracerebral hemorrhage (ICH) has the highest mortality rate among stroke subtypes, accounts for 15% to 20% of all stroke types, and has an increased incidence in elderly patients^1^^–^ 4. Acute ICH due to a large intracranial hematoma is associated with high morbidity and mortality, as it can lead to primary brain injury through the destruction of brain tissue and the high intracranial pressure (ICP) that results from the large hematoma^5^^,^ 6. Previous studies revealed that craniotomy for hematoma evacuation is an effective therapy for limiting primary brain damage and decreasing ICP after ICH, which is of substantial interest^6^^–^ 8. However, craniotomy for hematoma evacuation shows no clinical benefit to patients, no improvement in long-term outcomes, and rarely affects neurological recovery^9^. Increasing evidence shows that red blood cell debris, hemoglobin, its degradation products, and blood components trigger secondary brain injury following ICH and contribute to a series of damaging events, including neuroinflammation, brain edema, oxidative stress, blood-brain barrier (BBB) damage, and neuron death^10^^–^ 12, which can be reversed^13^^,^ 14. According to the previous studies^15^^–^ 17, inhibition of oxidative stress, decreases mitochondrial apoptosis, can improve neurological function, and decreases cerebral edema after ICH in mice. Currently, the neuroprotective effect of the inhibition of necroptosis and neuroinflammation remains unclear.

Ulinastatin (UTI) is a serine protease inhibitor with a molecular weight of 67,000 purified from human urine. The main pharmacological activities of UTI were anti-inflammatory, immune regulation, and organ protection^18^^,^ 19. A drug widely used to treat acute inflammatory disorders such as sepsis, ischemia-reperfusion injury, and antiapoptotic actions^20^. He^21^ reported that UTI may be of value for the inhibition of postoperative increased inflammatory agents and most likely provided pulmonary protective effects in cardiac surgery by meta-analysis of 15 randomized controlled trials. Additionally, in recent studies with animals, UTI had been reported that can alleviate early brain injury, cerebral ischemia-reperfusion injury, and permeability of the blood-brain barrier in the animal transient middle cerebral artery occlusion (tMCAO) model^22^^–^ 24. However, the effects of UTI on the EBI in the acute phase of ICH are not clear, and their association with levels of apoptotic molecules and oxidative stress remains to be elucidated.

Necroptosis is a newly discovered pathway of regulated necrosis, a caspase-independent programmed cell death mechanism that requires the proteins RIPK3 and MLKL and is induced by death receptors^25^. Increasing evidence suggests that necroptosis plays a critical role in central nervous system diseases, including traumatic brain injury^26^^–^ 28, ICH^29^^,^ 30, ischemic stroke^31^, amyotrophic lateral sclerosis, Parkinson’s disease, and Alzheimer’s disease^32^. The most upstream signaling activity required for the induction of necroptosis is the activation of a TNF ligand family member (e.g., protein kinase function of receptor-interacting protein kinase-1 [RIPK1] and mixed lineage kinase domain-like [MLKL]); RIPK1 activation leads to necroptosis through the formation of a RIPK1–RIPK3–MLKL complex^33^. Zhang^34^ also reported that selenium can prevent necroptosis by restoring antioxidant functions and blocking the MAPK/NF-κB pathway in the chicken spleen. Necroptosis is common in early brain injury^35^ and may be an effective mechanism of ICH.

In the present study, a mouse ICH model was constructed to study the effects of UTI on EBI and explored the crosstalk between oxidative stress and neuroinflammation. The mechanism by which the MAPK/NF-κB signaling pathway may regulate this process was also explored.

## Methods

All animal experiments performed in this study complied with the National Institutes of Health guidelines for the handling of laboratory animals and were approved by the Ethics Committee of the Wuxi Medical College of Anhui Medical University (YXLL-2020-049).

### Animal ICH model

A total of 45 healthy adult C57BL/6J mice were randomly assigned to the Sham group, SAH group, and SAH+UTI group. All experiments were conducted on healthy adult male C57BL/6J mice (22–25 g) (Anhui Medical University, Hefei, China). The mice were housed in animal care facilities on a 12 h light/dark cycle and had free access to food and water.

The ICH mouse model was generated based on a previously described protocol involving autologous blood injection^36^. Briefly, male C57BL6/J mice were anesthetized by intraperitoneal injection of 50 mg·kg^-1^ pentobarbital sodium and placed in a prone position with a stereotactic head frame. The rectal temperature was kept at 37 ± 0.5 °C during the operation using a heating pad. An artificial tear ointment was used to protect the eye from injury during surgery. A midline scalp incision was made, and a cranial burr hole with a 1-mm diameter was made at the following coordinates relative to bregma: 0.2 mm posterior, 2.2 mm lateral to bregma, and 3.5 mm below the dura. A total of 30 μL of autologous blood without anticoagulation was collected from the caudal artery and rapidly injected into the basal ganglia through the burr hole via the 26-gauge needle of a 10-μL Hamilton syringe. First, 5 μL of arterial blood was injected at a depth of 2.8 mm from the dura (injection speed: 3 μL·min^-1^). Five minutes later, the other 25 μL of blood was injected at a depth of 3.5 mm (injection speed: 3 μL·min^-1^). After the injection of autologous blood, the needle was kept in the brain for 10 min to prevent blood backflow along the needle tract. Finally, the hole was covered with medical bone wax. The animals in the Sham group received similar surgical procedures but were injected at the same site with an equal volume of sterile saline instead of blood.

### Drug administration

The UTI (Techpool Biochem, Guangdong, China) was stored at 4 °C and dissolved in 0.9% normal saline when it is used. A 10^4^ U·kg^-1^ UTI was administered by intraperitoneal injection before the onset of ICH^20^.

### Neurobehavioral assessment

The severity of brain injury was evaluated by determining neurological function 72 h after ICH using a previously described neurological grading system^37^. The scoring system consisted of motor, sensory, reflex, and balance tests. The neurological scores ranged from 0 to 18 points and were calculated by adding the scores together; all mice in each group underwent a behavioral assessment, and a higher score represented worse neurological function. All mouse behavior scores were recorded by the same independent observer who was blinded to the study groups.

### Brain water content measurement

The severity of brain edema was evaluated by measuring the brain water content using the standard wet-dry method, as previously reported^38^^–^ 40. The mice were sacrificed 72 h after ICH, and the entire brain was harvested and separated into the ipsilateral and contralateral cortices, ipsilateral and contralateral basal ganglia, and cerebellum (wet weight). Then, brain specimens from each group were dehydrated at 105 °C for 24 h to acquire the dry weight. The percentage of brain water content was equal to (wet weight – dry weight)/wet weight × 100%.

### Evans blue extravasation

Evans blue extravasation was performed as previously described^41^. Briefly, mice were anesthetized by pentobarbital sodium (50 mg·kg^-1^) injection 48 h after ICH. Evans blue dye (2%, 5 mL·kg^-1^; Sigma–Aldrich, St. Louis, MO, USA) was injected into the left femoral vein over 2 min and circulated for 60 min. Then, the mice were sacrificed with 100 mg·kg^-1^ sodium pentobarbital via intraperitoneal injection and with phosphate-buffered saline (PBS) intracardial perfusion. The brains were removed and quickly divided into the left and right cerebral hemispheres, weighed, homogenized in saline, and centrifuged at 15,000 g for 30 min. Subsequently, the resultant supernatant was added with an equal volume of trichloroacetic acid, incubated overnight at 4 °C, and centrifuged at 15,000 g for 30 min. Next, the resultant supernatant was collected and spectrophotometrically quantified at 610 nm for Evans blue dye.

### Cytokine measurements

The ipsilateral cortex tissue in animals and ICH patients’ serum were collected and detected. The levels of IL-1β (cat. No. ab197742; Abcam), IL-6 (cat. No. ab222503; Abcam), TNF-α (cat. No. ab208348; Abcam), and NF-κB (cat. No. ab176663; Abcam) were measured by the enzyme-linked immunosorbent assay (ELISA), according to the manufacturer’s instructions.

### TUNEL staining

A TUNEL assay was conducted to assess neuronal death in the hippocampus. The TUNEL reaction mixture (50 μL) was added to each sample, and the slides were incubated in a humidified chamber for 60 min at 37 °C in the dark. The slides were then incubated with DAPI for 5 min at room temperature in the dark to stain the nuclei, followed by imaging with a fluorescence microscope. The procedure was performed with a TUNEL staining kit according to the manufacturer’s instructions. A negative control (without the TUNEL reaction mixture) was used. The cell count was confirmed in four randomly selected high-power fields, and the data obtained from each field were averaged.

### Western blot analysis

Western blot analyses were performed as previously described^38^. Briefly, cerebral cortex samples were collected, homogenized, and separated by sodium dodecyl sulfate-polyacrylamide gel electrophoresis on 10% polyacrylamide gels. A BCA Protein Assay Kit (Beyotime) was used to measure protein concentrations with the bicinchoninic acid method. After separation, protein samples were transferred onto immobilon nitrocellulose membranes. The membranes were blocked with 5% nonfat milk at room temperature for 1 h. The membranes were then incubated with the following primary antibodies overnight at 4 °C: rabbit anti-β-actin (1:1000, rabbit polyclonal, Abcam, ab8227), rabbit anti-RIP1 (1:1,000; rabbit polyclonal; Abcam; cat. No. ab106393), rabbit anti-RIP3 (1:1,000; rabbit polyclonal; Abcam; cat. No. ab62344), phospho-p38 (CST, No. 4551, 1:2000), rabbit anti-NF-κB (1:1000, rabbit monoclonal, Abcam, ab32360). After washing the membranes with TBST three times, HRP-conjugated goat antirabbit IgG or goat antimouse IgG secondary antibodies (1:5000) were applied, and the membranes were incubated with the secondary antibodies at room temperature for 1.5 h. The protein bands were detected using a Bio-Rad imaging system (Bio-Rad, Hercules, CA, USA) and quantified with ImageJ software.

### Quantitative real-time polymerase chain reaction (PCR)

Quantitative real-time PCR analysis was performed as previously indicated^42^. Total RNA was extracted from either cell cultures or hippocampal brain samples using TRIzol Reagent (Gibco; Thermo Fisher Scientific, Inc., Waltham, MA, USA) according to the manufacturer’s instructions. Then, RNA was reverse transcribed to complementary DNA (cDNA) using the RevertAid First Strand cDNA Synthesis Kit (K1622; Thermo Fisher Scientific Inc., Rockford, IL). MAPK and Nrf2 mRNA levels in each sample were measured by qPCR using SYBR Green Master Mix (Toyobo Co., Ltd., Osaka, Japan). GAPDH was used as an internal control. The qPCR thermocycling conditions were as follows: 45 °C (2 min) and 95 °C (10 min), followed by 40 cycles of denaturation at 95 °C (15 s), annealing at 60 °C (1 min), and extension at 72 °C (1 min). All samples were analyzed in triplicate. The target genes and the specific primers are the following:

NF-κB (forward, 5’- ATCACGAGCCCTGAAACCAA-3’; reverse, 5’-GGCTGCAAAATGCTGGAAAA-3’),
MAPK (forward, 5’- TGTGTTCACCCCTGCCAAGT-3’; reverse, 5’-GCCCCCGAAGAATCTGGTAT-3’)
GAPDH (forward, 5’- ATGGGTGTGAACCACGAGA-3’ and reverse, 5’-CAGGGATGATGTTCTGGGCA-3’)

### Statistical analysis

The data are reported as the means and standard deviation (SD). The SPSS 14.0 (SPSS, Chicago, IL, USA) and GraphPad Prism 6 (GraphPad Software, San Diego, CA, USA) softwares were used for the statistical analyses. Student’s t-test was used if two groups were compared, and one-way analysis of variance (ANOVA) followed by Bonferroni’s post hoc test were used if two independent variables were compared. For all statistical analyses, differences were considered significant at p < 0.05.

## Results

### UTI alleviates neurological deficits and mortality after ICH

**Figure 1:**
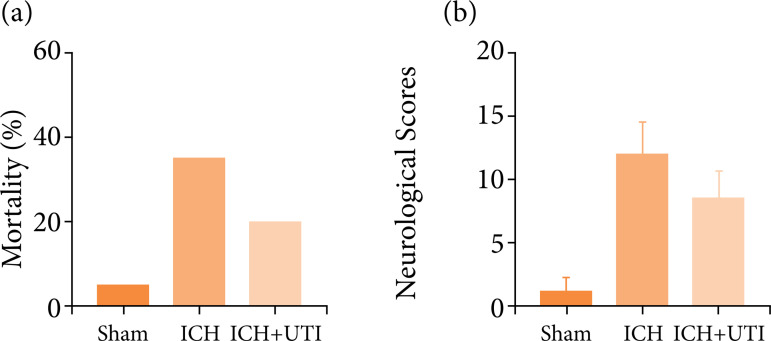
UTI alleviates neurological deficits and mortality after ICH. **(a)** Comparison of the mortality between the three groups, the mortality increased significantly after ICH, Mortality rates in the Sham group (5%), ICH group rate (35%)1, the ICH + UTI group rate (15%); **(b)** Neurological scores of mice in the sham group, ICH group, and ICH group treated with UTI at 72 h (n = 10, ^*^p < 0.05 vs. Sham; ^#^p < 0.05 vs. ICH; ANOVA; means ± SD).

The effect of UTI treatment on long-term neurological damage parameters was evaluated including mortality rates and neurological scores. As shown in Fig. 1, mortality rates (Fig. 1a) and neurological score (Fig. 1b) in various groups, including Sham, ICH, ICH + UTI. ICH significantly increased the mortality rates. It was alleviated by the UTI treatment, while no significant difference. Similar results were obtained for neurological scores, which were decreased significantly after ICH, and UTI administration significantly improved neurological function.

### UTI alleviate brain edema and BBB permeability after ICH

**Figure 2:**
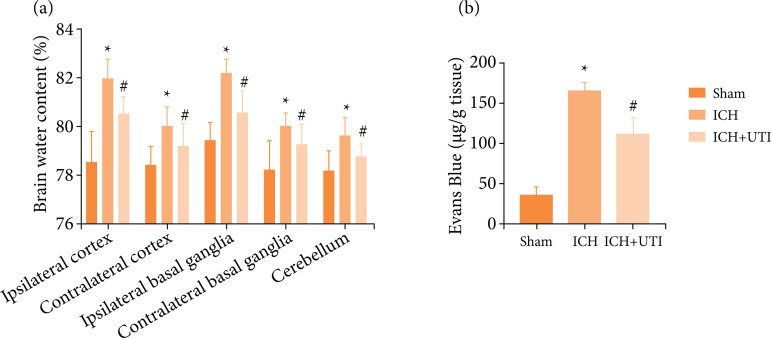
UTI alleviate brain edema and BBB permeability after ICH. **(a)** UTI alleviates brain water content after ICH; **(b)** UTI alleviates BBB permeability after ICH (n = 5, ^*^p < 0.05 vs. Sham; ^#^p < 0.05 vs. ICH; ANOVA; means ± SD).

To clarify the EBI after ICH, brain water content by the wet-dry method at 72 h after ICH was used to evaluate brain damage. The results showed that ICH increased the brain water content significantly, which was alleviated after UTI treatment (Fig. 2a). Similar results in BBB permeability, which were increased significantly after ICH, and UTI administration can significantly alleviate (Fig. 2b). Hence, UTI treatment markedly improves BBB permeability and alleviated brain edema at 72 h.

### UTI alleviates neuronal necroptosis after ICH

**Figure 3:**
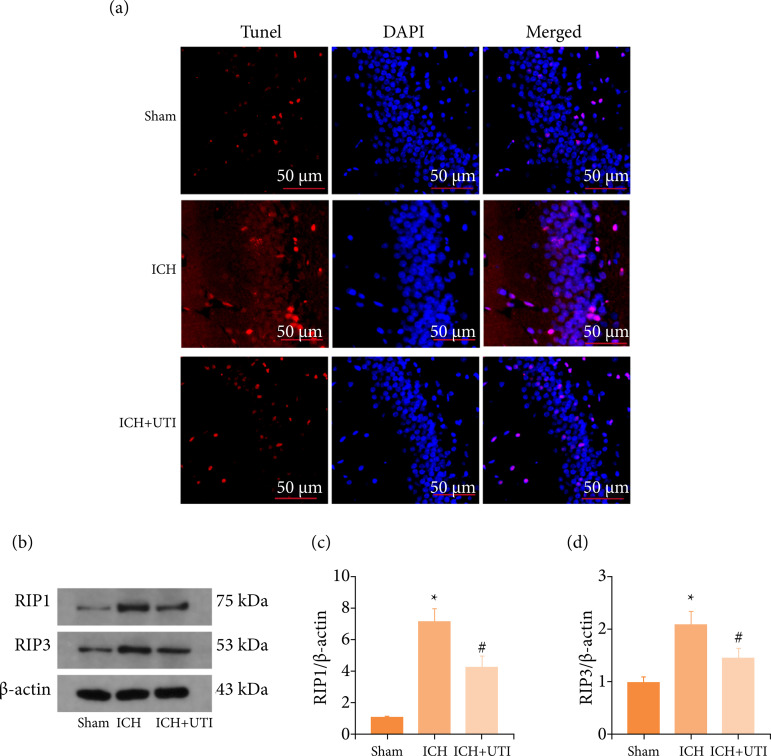
UTI alleviates neuronal necroptosis after ICH. **(a)** TUNEL staining showed that UTI alleviated neuronal apoptosis in the hippocampus at 72 h after ICH, and representative images of apoptotic neurons are shown. Scale bar = 50 μm; **(b)** Levels of RIP1 and RIP3 in the brain cortex of mice after TBI were determined using Western blotting; **(c)** Quantification of RIP1 levels in the brain cortex relative to β-actin, the loading control; **(d)** Quantification of RIP3 levels in the brain cortex relative to β-actin. DAPI, 4’,6-diamidino-2-phenylindole; SAH, subarachnoid hemorrhage; TUNEL, terminal deoxynucleotidyl transferase dUTP nick end labeling.

TUNEL assay was used to evaluate the level of cell death in ICH mice treated with and without UTI at 72 h after model construction. The results revealed more hippocampal neuronal death after ICH, and UTI decreased neuronal apoptosis (Fig. 3). Based on these results, UTI exerts neuroprotective effects after ICH. The expression levels of necroptosis-related protein were detected by western blotting (Fig. 3b). The results of western blotting also indicated that UTI can reduce the expression levels of necroptosis-related protein RIP1 and RIP3 (Fig. 3 c-d). These results demonstrate that UTI has neuroprotective effects after ICH.

### UTI alleviates neuroinflammation after ICH

**Figure 4:**
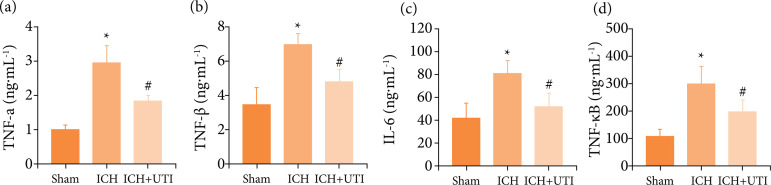
UTI alleviates neuroinflammation after ICH. UTI significantly reduced hippocampal **(a)** TNF-α; **(b)** interleukin-1β (IL-1β); **(c)** IL-6; and **(d)** NF-κB levels at 72 h after ICH (n = 5, ^*^p < 0.05 vs. Sham; ^#^ p < 0.05 vs. ICH, ANOVA; means ± SD).

As previous studies^34^^,^ 40 have identified a vital role for neuroinflammation in EBI after ICH, increased neuroinflammation aggravates EBI. The inflammatory complex induces the secretion of proinflammatory cytokines, including IL-1β, IL-6, and TNF-α, and the subsequent activation of proinflammatory signaling through NF-κB to initiate pyroptosis. Therefore, the hippocampal levels of IL-1β, IL-6, TNF-α, and NF-κB were measured using ELISAs. The levels of the proinflammatory cytokines were increased significantly after ICH, while the levels of proinflammatory cytokines decreased significantly after UTI treatment (Fig. 4 a-d). Hence, these results suggested that UTI exhibited potent anti-inflammatory activity against ICH-induced neuroinflammation.

### UTI regulates necroptosis and neuroinflammation by modulating the MAPK/NF-κB signaling pathway after ICH

**Figure 5:**
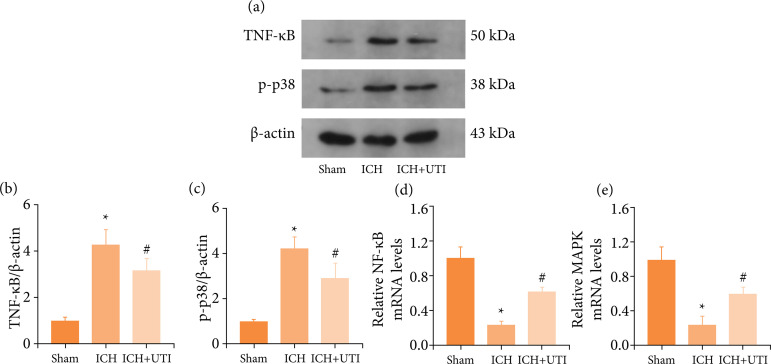
UTI regulates necroptosis and neuroinflammation by modulating the MAPK/ NF-κB signaling pathway after ICH. **(a)** Levels of MAPK, and NF-κB in the brain cortex of mice after ICH were determined using Western blotting; **(b)** Quantification of NF-κB levels in the brain cortex relative to β-actin, the loading control; **(c)** Quantification of MAPK levels in the brain cortex relative to β-actin; **(d)** Levels of NF-κB mRNA in the brain of ICH mice weremeasured by real-time PCR; **(e)** Levels of MAPK mRNA in the brain of ICH mice were measured by real-timePCR (n = 5, Data are presented as the mean ± SD, ^*^p < 0.05 vs. Sham; ^#^p < 0.05 vs. ICH).

MAPK/NF-κB was a core signaling pathway of necroptosis and neuroinflammation^43^^,^ 44. Whether the neuroprotection of UTI regulates necroptosis and neuroinflammation was explored by modulating the MAPK/NF-κB signaling pathway after ICH. The levels of the MAPK and NF-κB protein were detected by performing western blotting (Fig. 5a). The levels of MAPK and NF-κB were increased significantly in the ICH group and decreased after UTI administration (Fig. 5 b-c). Additionally, real-time PCR also demonstrated a similar result (Fig. 5 d-e). Thus, these results showed that neuroprotection of UTI may be by regulating the MAPK/ NF-κB signaling pathway.

### UTI decrease the levels of the proinflammatory cytokine in ICH patients

**Figure 6:**
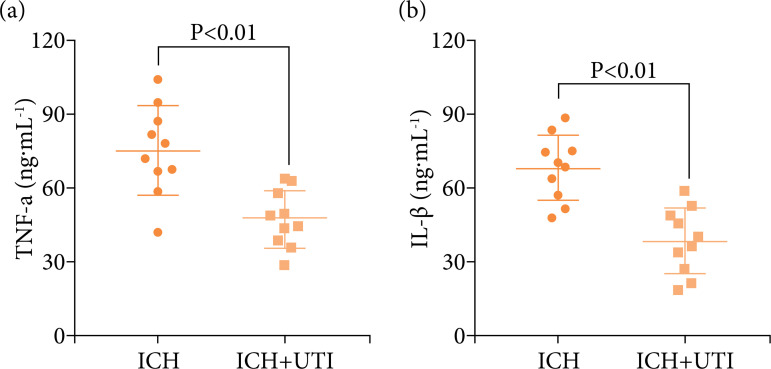
UTI alleviates neuroinflammation in ICH patients. UTI significantly reduced ICH patients’ serum **(a)** TNF-α; **(b)** interleukin-1β (IL-1β) levels at 72 h after ICH (n = 10, p < 0.01, ANOVA; means ± SD).

To verify the antineuroinflammation in ICH patients, the expression levels of serum IL-1β, IL-6, TNF-α, and NF-κB in ICH patients were evaluated. The serum levels of IL-1β, IL-6, TNF-α, and NF-κB were measured using ELISAs. The levels of the proinflammatory cytokines were increased significantly after ICH, while the levels of proinflammatory cytokines decreased significantly after UTI treatment (Fig. 6 a, b), the results were similar to the animals.

## Discussion

**Figure 7:**
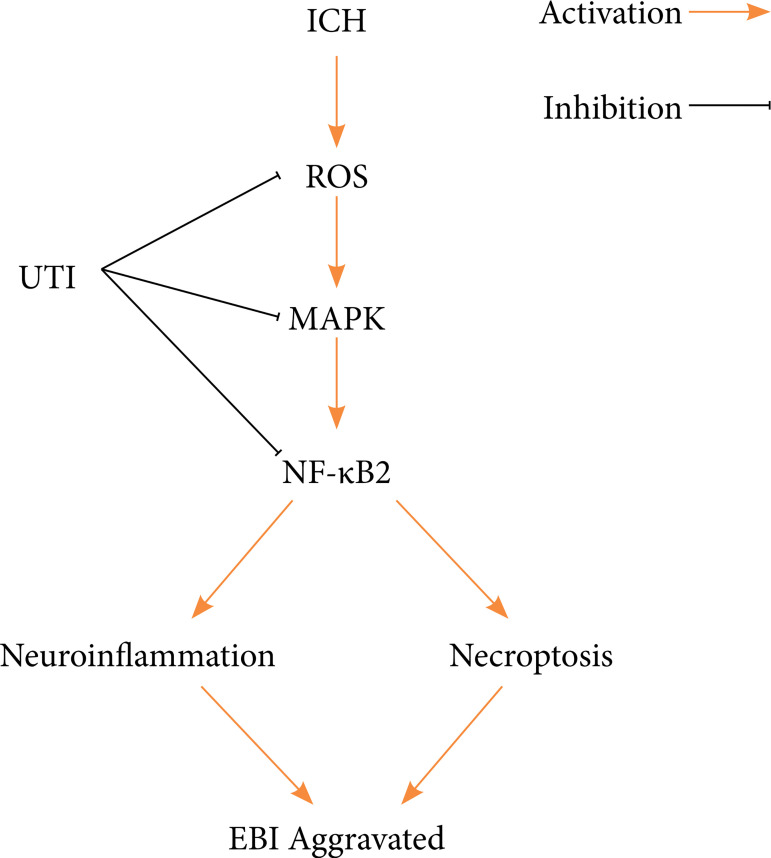
A diagram of the proposed model explaining the observations of MAPK/NF-κB -mediated regulation of necroptosis, and neuroinflammation after ICH and potential mechanisms underlying the effect of the UTI intervention.

Here, the therapeutic potential of UTI to alleviate early brain injury was evaluated in a mouse model of ICH. As shown in the present study, UTI is a neuroprotective agent that attenuates early brain injury following ICH. It showed that UTI (1) improves neurological dysfunction after ICH, (2) alleviates brain damage in a mouse ICH model, (3) relieves neuroinflammation after ICH and then decreases inflammatory damage in the brain, and (4) prevents necroptosis after ICH and alleviates neuronal death; (5) the antinecroptosis and antineuroinflammation effects of UTI may be related to the MAPK/NF-κB signaling pathway (Fig. 7).

UTI was a 67 kDa glycoprotein purified from the urine of healthy humans, nonspecific protease inhibitor, a urinary trypsin inhibitor, which was used to treat acute inflammatory disorders, sepsis, toxic shock, and hemorrhagic shock^45^^,^ 46. Recent studies had demonstrated that UTI can alleviate cerebral ischemia-reperfusion injury by regulating inflammation and oxidative stress^22^^,^ 47. However, the neuroprotection of UTI in ICH was unclear, and lacked related clinical studies. Liu^19^ reported that UTI can decrease the brain water content and blood-brain barrier permeability significantly after ICH, maybe through decreased activation of the astrocytes and ET-1, inhibited the expression of proinflammatory VEGF and MMP-9. Another study also reported that UTI can attenuate brain edema after male Sprague-Dawley rats ICH, the preliminary molecular mechanism may be through the decrease of the expression level of aquaporin-4 (AQP4), and proinflammatory cytokines including IL-1β and TNF-α as well as activity of NF-κB^48^. The present study also demonstrated that UTI can alleviate brain edema, improve neurological function, relieve neuroinflammation response, and decrease hippocampal neuronal damage and necroptosis. Yang^49^ reported that necroptosis and neuroinflammation were important mechanisms to lead EBI after ICH, and inhibition of these mechanisms can improve neurological outcomes, and reduce neuronal damage. Pan^50^ also indicated that antinecroptosis chemical necrostatin-1 can suppress apoptosis and autophagy in mice ICH model. Similar results were also demonstrated and the potential mechanism may be through RIP1/RIP3/MLKL pathway^51^^,^ 52.

As the pharmacological action of UTI was more complex, included anti-inflammatory, immune regulation, and organ protection^18^^,^ 19. The mechanism of neuroprotection of UTI after ICH also was multiple and complicated. Li^23^ also demonstrated that UTI can protect the brain against ischemic injury, the potential molecular mechanism may be through to restore the BBB permeability by decreased expression of MMP-9 and increased ZO-1 and occludin proteins expressions. The mechanisms and molecules regulating necroptosis and neuroinflammation were very complex. The results in the present study, UTI also can decrease the expression levels of MAPK and NF-κB, then alleviate the activation of necroptosis and neuroinflammation. Activated p38 MAPK could facilitate the disassociation of Nrf2 from Keap1 to initiate the transcription of several antinecroptosis, antiferroptosis, and antioxidant genes, such as HO-1, quinine oxidoreductase-1 (NQO1), and glutathione peroxidase 4 (GPX4)^6^^,^ 53. Li^54^ also reported that UTI can improve neurological function, and alleviate brain edema and infarct volume by decreasing the expression of TLR4 and NF-κB in the tMCAO model. Cui^48^ also confirmed that the activity of IL-1β, TNF-α, and NF-κB was inhibited by UTI treatment in traumatic brain injury. ICH leads to intracellular reactive oxygen species accumulation and decreases the expression levels of TLR4, and the TLR4/NF-κB/p65 signaling pathway also directly regulates neuroinflammation and necroptosis. In the present study, it was also observed that UTI can alleviate EBI after ICH through regulating crosstalk between oxidative stress and neuroinflammation, the potential mechanism may be mediated MAPK/NF-κB signaling pathway. The specific mechanism remains unclear, and other potential molecular mechanisms may coexist and play a synergistic role. Therefore, further research is needed to explore these mechanisms. In addition, this experiment was performed in mice, and debate persists regarding whether the treatment is effective in humans. In the future, the clinical effect of UTI on patients with ICH will be further explored.

## Conclusions

In summary, this study provided evidence that necroptosis and neuroinflammation are emerged as important cellular regulatory mechanisms, and contributed to EBI after ICH. In this study, the UTI-mediated regulation of necroptosis and neuroinflammation by the MAPK/NF-κB pathway and provided a new idea to explore the biological effects and mechanisms underlying the antinecroptosis, anti-inflammatory, and neuroprotective properties of UTI.
